# Ventilatory ratio: validation in an *ex vivo *model and analysis in ARDS/ALI patients

**DOI:** 10.1186/cc9599

**Published:** 2011-03-11

**Authors:** P Sinha, K Corrie, A Bersten, JG Hardman, N Soni

**Affiliations:** 1 Chelsea and Westminster NHS Foundation Trust, London, UK; 2 Queen's Medical Centre, Nottingham, UK; 3 ANZICS CTG, Flinders Medical Center, Adelaide, Australia

## Introduction

Several indices exist to monitor adequate oxygenation, but no such index exists for ventilatory efficiency. The ventilatory ratio (VR) is a simple tool to monitor changes in ventilatory efficiency using variables commonly measured at the bedside [1]:

$$\text{VR}\;=\;\frac{{\dot{V}}_{E_{measured}}\times P_{a_{co_{2\;measured}}}\;}{{\dot{V}}_{E_{predicted}}\times P_{a_{co_{2\;predicted}}}\;}$$

**Figure 1:**
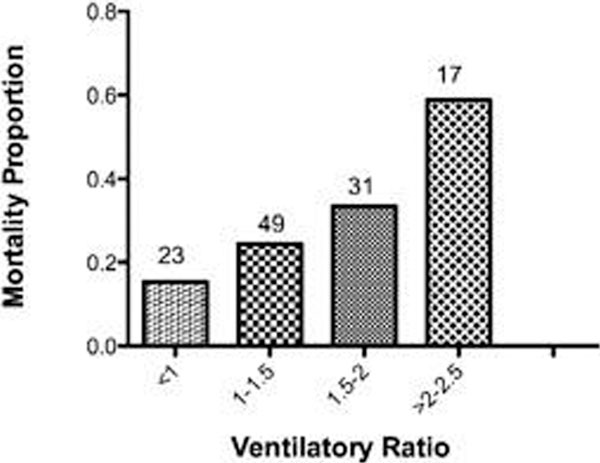
**Chi-squared test for trends *P *= 0.0015**.

See Figure 1 overleaf (where predicted values are VE 100 ml/kg/minute and PaCO_2 _5 kPa).

## Methods

The Nottingham Physiology Simulator (NPS), a validated computational model of cardiopulmonary physiology [2], was used to validate the ability of VR to reflect ventilatory efficiency *ex vivo*. Three virtual patients were configured, representing healthy lung, ARDS and COPD. VR was calculated while minute ventilation, ventilation rate and VCO_2 _were each varied in isolation. The clinical uses of VR were then examined in a database comprising 122 patients with ALI and ARDS [3]. Standard respiratory data and VR values were analysed in all patients.

## Results

The NPS model showed significant correlation between VR and physiological deadspace fraction (Vd/Vtphys) at constant VCO_2 _(*P *< 0.001, *r *= 0.99). Similarly, VCO_2 _had a linear relationship with VR at constant Vd/Vtphys. Across the various ventilatory configurations the median values and ranges of calculated VR for the three patients were as follows: normal patient VR 0.89 (0.61 to 1.36), COPD 1.36 (0.95 to 1.89) and ARDS 1.73 (1.2 to 2.62). In the ALI/ARDS database the range of values for VR was 0.56 to 3.93 (median 1.36). Patients with ARDS had a significantly higher VR in comparison with patients with ALI (1.44, 1.25 to 1.77 vs. 1.25, 0.94 to 1.6, *P *= 0.02). VR was significantly higher in nonsurvivors as compared with survivors (1.7 ± 0.64 vs. 1.45 ± 0.56, *P *< 0.03). There was poor correlation between PaO_2_/FiO_2 _ratio and VR in the population (*r *= -0.32, 95% CI = -0.47 to -0.15).

## Conclusions

*Ex vivo *modling shows that VR can be simply and reliably used to monitor ventilatory efficiency at the bedside. VR is influenced by changing CO_2 _production and deadspace ventilation. As a clinical tool it is a predictor of outcome and is independent to PaO_2_/FiO_2 _ratio.
